# Knowledge and Attitudes about the Flu Vaccine among Pregnant Women in the Valencian Community (Spain)

**DOI:** 10.3390/medicina55080467

**Published:** 2019-08-11

**Authors:** Noelia Rodríguez-Blanco, José Tuells

**Affiliations:** 1 Department of Obstetrics and Gynaecology, Hospital Universitario del Vinalopó, Spain C/Tonico Sansano Mora, 14, 03293 Elche, Spain; 2 Department of Nursing Universidad CEU Cardenal Herrera. Plaza Reyes Católicos, 19, 03204 Elche, Spain; 3 Cátedra Balmis de Vacunología. University of Alicante. Campus de San Vicente Raspeig. Ap.99, E-03080 Alicante, Spain

**Keywords:** influenza, pregnancy, immunization, vaccine coverage, acceptability, vaccine hesitancy

## Abstract

*Background and Objectives:* To describe the knowledge and attitudes related to the acceptance of the flu vaccine during pregnancy in women, from two Health Departments of the Valencian Community (VC), during the 2015–2016 season, after receiving prenatal care. *Materials and Methods:* A prospective observational study was conducted during the annual vaccine season of women ascribed to prenatal care. A midwife offered flu vaccine advice and afterwards conducted a telephone poll of a representative sample, in order to find out the reason for accepting or rejecting the vaccine. *Results:* Of the 1017 expectant women who received advice about the vaccine, 77.4% (95% CI: 74.8–79.9%) declared their intention to vaccinate. After the recommendation, the vaccine coverage was 61.6%, with a percentage of accordance of 98.8% (95% CI: 98.0–99.6%) between the coverage declared and the Nominal Vaccination Registry (NVR) of the VC. Additionally, 67.2% of the expectant women were interviewed (*n* = 683). Most were aware of the recommendation and identified the health center and the midwife as the main sources of information. The internet was a consistent source in favor of vaccination 80.8% (*n* = 42). The obstetric variables (risk during the pregnancy, end of pregnancy, and feeding the newborn) did not have a statistically significant relationship with the vaccination. The women declared a high adherence to the vaccinations present in the child vaccination calendar, but rejected (31.3%) the flu vaccine, as they had not received it previously and did not want it because of their expectant state. *Conclusions:* The women positively evaluated the effectiveness and safety of the vaccines. However, with the flu vaccine, “not being previously vaccinated” and the “doubts about its safety” represented more than half of the reasons put forth for its rejection. Ensuring that the flu vaccine is perceived as more effective and acceptable through the messages directed towards the expectant mothers, directly through the midwives or through the communication media and social networks, will result in an increase of vaccine coverage.

## 1. Introduction

Recommendations for vaccines are constantly expanding, given the addition of new susceptible groups to the population that need immunization protection, and the development of greater evidence about the safety and benefits of vaccines [1].

Pregnant women and children below one year-old are the two collectives that are at most risk of being severely affected by transmissible diseases, and this is the reason why the World Health Organization (WHO) has recommended vaccination against the flu in any trimester of pregnancy, including them as a group with a high-risk of serious diseases [2].

To achieve a successful immunization program, it is necessary for the population to have confidence on the benefits and safety of the vaccines. The delay of vaccination or its rejection (vaccine hesitancy) is multifactorial, and is related to complacency, the difficulty of accessing the vaccines, or the lack of confidence towards a specific vaccine [3,4,5].

At present, vaccine coverage and the acceptance of vaccines during pregnancy is sub-optimal, and this is due, overall, to the fear of the adverse effects and the lack of information or advice from health professionals. Thus, the objectives set forth by the Healthy People 2020 initiative for influenza, in the US, point to a 80% coverage as desirable. In this country and in the rest of the world, coverage does not exceed 50% of flu vaccination for pregnant women and the registration of vaccination activities is not homogeneous [6,7,8,9]. In Spain, vaccine advice was introduced into any trimester of pregnancy starting in 2013, and from that time onwards, an overall coverage of around 27.6% was achieved and in the last 2018-2019 season of 38.5%. [10,11].

Health professionals, especially those in primary care, offer vaccine advice to expectant women during the flu season campaign period [12]. Children of vaccinated mothers receive passive immunization through placental transfer of antibodies and breastfeeding [13,14,15]. Continuous efforts are needed to increase the knowledge about the safety of the vaccine among pregnant women. In this context, health professionals and the communication media play an important role [16,17] to ensure adequate prenatal attention [18,19].

Efforts are also being made that are geared towards improving the vaccine itself. Mothers’ perceptions of the safety and effectiveness of the influenza vaccination are related to the achievement of a longer-lasting effectiveness (it is estimated that it decreases three months after its administration), a greater matching with the annual epidemic trends, and the perception that flu is not dangerous during pregnancy [3,20,21].

The objective of this research was to estimate the degree of acceptance of the flu vaccine by evaluating its relation with obstetric, sociodemographic, and temporal variables, and analyzing the reasons behind the rejection during the pregnancy of a sample of expectant women in two health departments, Torrevieja (TV) and Elche-Crevillente (EC) from the Valencian Community (VC, Spain).

## 2. Materials and Methods

### 2.1. Study Design

A prospective observational study, directed towards expectant women who were tended to during the monitoring of their pregnancy, was conducted during the 2015–2016 flu season campaign (15th October 2015, to 31st January 2016) in two health departments (TV and EC), which were comprised by 10 primary care health centers. These two departments provide health coverage to a total of 320,000 inhabitants (170,000 in TV and 150,000 in EC).

### 2.2. Participant Recruitment and Informed Consent

Initially, a training session was provided for the midwives from the 10 primary health centers. They were trained on flu vaccine advice and a registry was created for the gathering of the study variables. The expectant women were selected in a consecutive manner, according to their visit, with an appointment to the midwife’s office. The ones who declined to participate due to a language barrier, those who had suffered the death of the fetus, or had a contraindication for receiving the flu vaccine, were excluded. The study variables included in the registry included sociodemographic data and obstetric characteristics of the expectant women; the health department (TV/EC), country of origin (Spain/Not Spain); age (≤24, 25–29, 30–34, 35–39, ≥40); previous pregnancies (1, 2, ≥3); number of abortions (0, 1, 2), current pregnancy trimester (1T, 2T, 3T), and month of visit to the midwife (October, November, December, January); and the intention or predisposition for becoming vaccinated with the flu vaccine.

After obtaining the sample of the expectant women who complied with the criteria, a second phase of the study began. This included a telephone interview and the review of the obstetric data of the Medical History. Through a structured questionnaire designed ad hoc, the women who were in the postpartum stage during the period ranging from 1 June to 30 September, 2016, were interviewed through the phone. The questionnaire collected variables about their knowledge, sources of information, place, and beliefs regarding the flu vaccine. Those who were not vaccinated declared their reasons for rejection. Likewise, the obstetric variables obtained from the Medical History were associated with vaccination or non-vaccination before giving birth. The vaccination coverage obtained was compared and contrasted with the nominal registry of vaccines (NRV) of the VC.

The women polled were assured about the confidentiality and anonymity of the collected data, as well as the right to not answer the questions.

### 2.3. Data Analysis

The statistical analyses were performed through the SPSS program version 20.0. For all variables, the frequencies and percentages of their categories were calculated, as well as the confidence interval at 95% (95% CI). The vaccine coverage was calculated as the percentage of vaccinated women with respect to the total sample, and its 95% CI was found as well. The chi-square test was used to analyze the statistical significance of the differences in the vaccine coverage percentages between the variable categories.

### 2.4. Ethical Approval

The study took into consideration the ethical principles for medical research established in the current legislation and was approved by the Research Commission of the participating centers after their authorization (#134-14) by the Spanish Agency of Medicines and Health Products (AEMPS) on 26 October 2015.

## 3. Results

### 3.1. Sociodemographic and Obstetric Characteristics of the Sample

A total of 1017 expectant women received advice on the flu vaccine during a monitoring of their pregnancy, which was performed by the midwife. Of these, 69.2% were Spanish, and the average age was 30.4 ± 5.6. For 94.7% of the expectant women, the pregnancy was their second one, and 98.0% of them had not had a previous abortion.

During the 2015–2016 vaccination campaign, the midwife gave advice to the expectant women in all trimesters of the pregnancy (1T: 191, 2T: 243, 3T: 249). The month with the greatest number of recommendations recorded was November, which was at 68.4% (*n* = 467), and the one with the least number was January, which was at 3.1%. The intent to vaccinate was 77.4% (95% CI: 74.8–79.9%) during the visit to the midwife.

**Figure 1:**
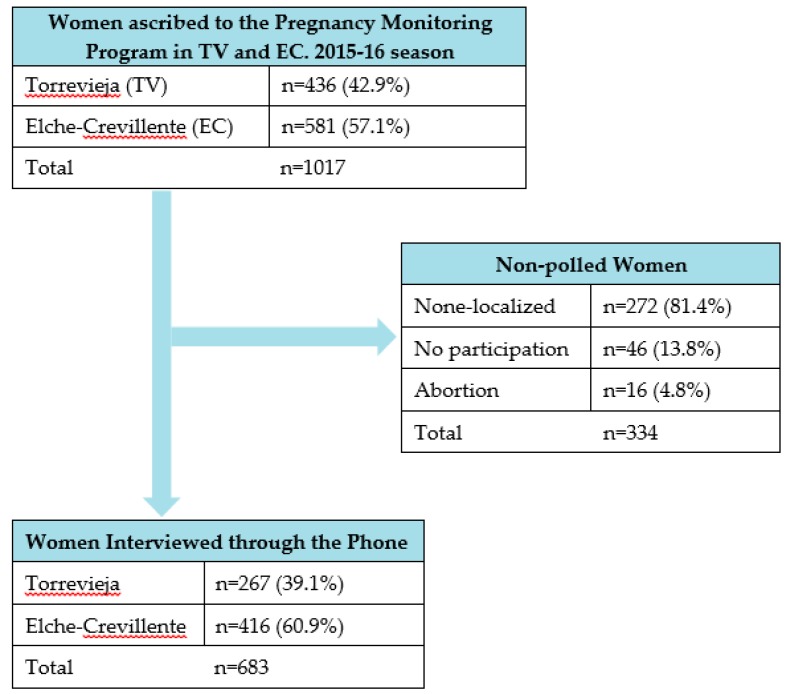
Flow diagram of the study.

A total of 683 (67.2%) post-partum mothers participated in the phone poll about knowledge, sources of information and reasons for vaccination, and they were distributed according to the health department as 267 (39.1%) in TV and 416 (60.1%) in EC. Those who were not polled due to different reasons, totaled at 334 (32.8%) (Figure 1).

### 3.2. Knowledge and Sources of Information about the Flu Vaccine

Of the 683 polled, the vaccination coverage was 61.6%. The expectant mothers who were also registered in the NVR obtained a high correlation with a Kappa value k = 0.974 (*p* < 0.001) with a percentage of agreement of 98.8% (95% CI: 98.0–99.6). Additionally, 91.7% (*n* = 626) was aware about the flu vaccine during pregnancy, and 64.2% (*n* = 402) were vaccinated. The primary health center was the place where most of the 68.4% (*n* = 467) obtained information about the vaccine, followed by friends and family, with a total of 12.2% (*n* = 83). In the group of the vaccinated expectant women, consultation from the Internet as a favorable source for vaccination was statistically significant, while for the non-vaccinated group, the lack of information was underlined, which was at 63.3% (*n* = 31).

Most of those polled, 92.2%, declared having received the recommendation to vaccinate, although 35.1% was not vaccinated. For 88.4%, the midwife was the most-active professional worker who recommended vaccination, followed by the family doctor (6.4%) (Table 1).

Obstetric variables for the pregnant women that have had a previous birth were collected from the medical history records of any of the two health departments (risk during the pregnancy, type of end of pregnancy, and feeding of the newborn after hospital discharge). These 628 women did not show significant differences in having received the flu vaccine or not (Table 2).

### 3.3. Beliefs about Vaccines in General and Their Relationship with the Flu Vaccine

The opinion on the vaccines in general was favorable or very favorable for 88.0% (*n* = 601, 95% CI: 85.4–90.6) of the expectant women and 98.5% (*n* = 673, 95% CI: 97.6–99.4) manifested having received all vaccines according to the vaccination calendar, throughout their lives, with both variables having a *p* < 0.01.

### 3.4. Reasons Declared for Rejecting the Flu Vaccine

The reasons for non-vaccination were, “not having previously received the flu vaccine”, 31.3% (*n* = 82); “fear of the adverse effects of the vaccine”, 20.6% (*n* = 54); and “the low confidence on the vaccine”, 17.2% (*n* = 45). Only 12.2% (*n* = 32) affirmed not vaccinating due to not receiving advice from the health professional.

**Figure 2:**
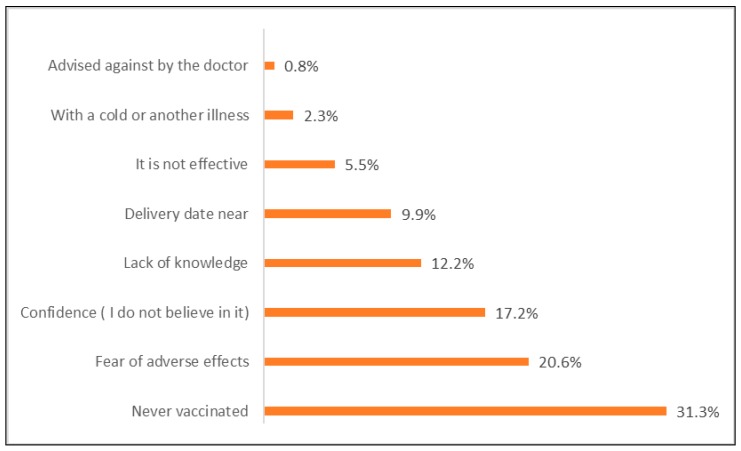
Reasons provided for not vaccinating.

Other reasons were also cited, such as ‘giving birth before the vaccination appointment’, 9.9% (*n* = 26) and the ‘belief that the flu vaccine is not effective’, 5.5% (*n* = 15) (Figure 2).

## 4. Discussion

The reasons that affected the decision to become vaccinated or not were analyzed in this research work. The intent to vaccinate in the initial sample (*n* = 1017) after the midwife’s advice was 77.4%, like the results from a study by Hu et al. [22]. The expecting women feel vulnerable and the health professionals play an important role in the entire process of mother–fetus health [23]. The Spanish health authorities, as well as that in other countries, are worried about the low vaccination coverage, lower than 40–50% [6,9,11,24]. The VC, one of the 17 Autonomous Communities of Spain and the place where the present research took place, obtained the greatest percentage of flu vaccine coverage of pregnant women, being 52% for the 2018–2019 season [11,25]. The vaccine coverage in this study, for the 2015–2016 season, reached up to 62%.

In light of the results obtained in this work, and after a literature review, we can attest that there are many factors, to a greater or lesser degree, related to vaccination that could be grouped into four categories—the setting, the pregnancy, the health professionals, and the fetus/newborn.

With regards to the setting, the fact that the vaccine is free [26], is administered at the doctor’s office itself [7,27], the use of reminders such as text messages [28], and the degree of effectiveness achieved in a season (that the vaccine strains forecasted in this campaign are the same as the ones that are circulating), are factors that are favorable for vaccination [21,29]. The need for an annual vaccination [20,30], and the decrease in the immunizations in the last months of the campaign, as observed in this work, are factors that have a negative effect. This “calendar effect”, where the professional’s recommendation decreases as the campaign moves forward, affects the final vaccination coverage [31]. The information was obtained by the expectant women in the primary education setting and through new technologies (Internet, social networks). As for the last point, it is important to evaluate their use and influence on the making of decisions about one’s and one’s descendants’ health [32,33].

With respect to pregnancy, statements such as “lack of confidence” or “not having been vaccinated ever” were reasons for rejecting the vaccine [5,34]. The women perceived the vaccination as something that was not necessary for the pregnancy [35]. It is fundamental that the health professionals are able to resolve worries and transmit confidence on the safety of the vaccines [19,36]. The association between abortions and vaccines against the flu from previous years could create a certain worry, although there are certain limitations for confirming it [37]. In spite of this, in the 2017–2018 season in the USA, the vaccination coverage descended from 53.6% to 35.6% [38]. As for the obstetric variables studied, significant data related to the vaccination was not obtained, although another study [39] has pointed to the association between a high-risk pregnancy and a greater vaccination rate. Another type of pre-pregnancy care (before conception or between pregnancies) could prevent possible infectious diseases that can affect the pregnant women, the fetus and the newborn, as long as they include an inspection of the vaccines, which should be given before the pregnancy, during the pregnancy, and at the post-partum stage [18,40]. The non-vaccinated pregnant women have a high risk of becoming sick from the flu and a greater risk of hospitalization [15,41,42].

Recommendation and counseling by a health professional is key for achieving a high vaccine coverage [11,19,34,35,36,38]. In our study, the main reason for not vaccinating was not the lack of recommendations by a professional, given that most of the expectant women had received it [43,44]. The midwife, in her monitoring and counseling role during the process of monitoring of the pregnancy, significantly increased the vaccine coverage [25,45]. The appearance of health alerts for professionals and users [46], as well as the handing out of leaflets at the office [47] or text messages [48], could be effective. In this work, the re-enforcement of the midwife about the benefits at the health center, and the information provided by the internet [49] were the most notable predictors for the correct vaccination.

As for the effect on the fetus/newborn, the vaccine is safe for the mother and child. The women have a smaller probability of having a complication during the pregnancy if they are vaccinated, and therefore, this is also true for the fetus [44,50,51]. The pregnant woman is given two vaccines during the pregnancy, for the flu and whooping cough, with the second one obtaining the highest percentages in Spain [25]. This could be due to the women identifying the child as the beneficiary of the whooping cough vaccine, and the mother being the beneficiary of the flu vaccine. Thus, this could be the reason why this last vaccine loses value or importance [52]. The health professionals must insist on explaining the benefits of the flu vaccine for the fetus, otherwise incomplete information is being provided.

The barriers related with the resistance to the vaccines are due to reasons that are psychological, contextual, sociodemographic, and physical in character [3,5,31]. It is important to understand the reasons for the behavior of rejection of this vaccine, as an opportunity for modifying said behaviors [34,53]. Health literacy must be in accordance with the culture and must adapt to the characteristics of the region. Communication by radio, television and the use of new technologies also improve vaccine coverage. [32,54,55].

As for the limitations of the study, the state of vaccination was declared by the women, and could be subject to recall bias or social desirability bias. Within the strengths, the obtaining of a sample size of sufficient size, with a high rate of participation is notable, as it facilitated the obtainment of the reasons for rejection even after recommendations by a professional, and the high agreement obtained between the vaccine coverage declared and the one registered in the NVR.

This research study can serve as the basis for the development of more effective strategies when providing health recommendations to women of childbearing age and the impact on health achieved with the alliance between professionals and new technologies.

## 5. Conclusions

This study clearly confirms that after the recommendation of the flu vaccination in any trimester of the pregnancy, the pre-disposition towards vaccination of the expectant women was very high. The vaccine advice should be maintained throughout the seasonal campaign. The women mentioned having received the vaccines included in the vaccination calendar throughout their lives and positively evaluated their effectiveness and safety. However, this was not the case for the flu vaccine, as the reasons “not having been vaccinated before” and “doubts about its safety” represented more than half of the reasons put forth for rejecting it. This fact ratifies the particular lack of predisposition towards it, not only by the expectant mothers, but also by some health professional collectives and the population in general. We should not forget that the WHO has declared that “vaccine hesitancy” is one of the 10 main world threats for public health [56].

This underlines the importance of the proactivity of the health professionals in the medical offices and the need to bring positive messages to the public sphere through social networks and the internet, two key elements for obtaining an optimal vaccination coverage.

## Figures and Tables

**Table 1:** Distribution of frequencies and percentages in women surveyed on the knowledge and sources of information about the flu vaccine.

		Vaccinated	Non-Vaccinated	Total	CI (95%)
Overall (n/%)		(421/61.6%)	(262/38.4%)	(683/100%)	
**Have you Heard about the flu vaccine?** *					
**Yes**		402 (64.2%)	224 (35.8%)	626 (91.7%)	(89.5–93.8)
**No**		19 (33.3%)	38 (66.7%)	57 (8.3%)	(1.1–15.4)
**Where did you obtain information about the flu vaccine during your pregnancy?**					
**Family–Friends**		50 (60.2%)	33 (39.8%)	83 (12.2%)	(5.1–19.2)
**Internet** *		27 (74.4%)	12 (25.6%)	39 (5.7%)	(0.0–12.9)
**Private Office**		42 (80.8%)	10 (19.2%)	52 (7.6%)	(0.4–14.8)
**Hospital**		9 (81.8%)	2 (18.2%)	11 (1.6%)	(0.0–9.0)
**Health Center**		15 (75.0%)	5 (25.0%)	20 (2.9%)	(0.0–10.2)
**No information** *		285 (61.0%)	182 (39.0%)	467 (68.4%)	(64.2–72.6)
**Media (press, radio, TV)**		18 (36.7%)	31 (63.3%)	49 (7.2%)	(0.0–14.4)
**Has anyone recommended vaccination to you?** *					
**Yes**		409 (64.9%)	221 (35.1%)	630 (92.2%)	(90.1–94.3)
**No**		12 (22.6%)	41 (77.4%)	53 (7.8%)	(0.5–15.0)
**Family Doctor**		31 (70.5%)	13 (29.5%)	44 (6.4%)	(0.0–13.6)
**Who**	**Midwife** *	391 (64.7%)	213 (35.1%)	604 (88.4%)	(85.8–90.9)
	**Nurse**	0 (0.0%)	1 (100%)	1 (0.1%)	(0.0–6.3)
	**Gynecologist**	6 (75.0%)	2 (25.0%)	8 (1.2%)	(0.0–8.7)
	**Family/Friend**	9 (42.9%)	12 (57.1%)	21 (3.1%)	(0.0–1.4)
	**No one** *	12 (23.1%)	40 (76.9%)	52 (7.6%)	(0.3–14.8)

* Statistically significant differences between the vaccinated and non-vaccinated (*p* < 0.05).

**Table 2:** Frequencies and percentage distribution of the declared flu vaccine and the obstetric variables from medical history.

		Vaccinated	Non-Vaccinated	Total	CI (95%)
Overall (n/%)		(378/60.2%)	(250/39.8%)	(628/100%)	
**Risk during pregnancy**	**Low**	343 (90.7%)	228 (91.2%)	571 (90.9%)	(88.5–93.2)
	**High**	35 (9.3%)	22 (8.8%)	57 (9.1%)	(1.6–16.6)
**End of pregnancy**	**Vaginal**	292 (77.2%)	194 (77.6%)	486 (77.4%)	(73.7–81.1)
	**Caesarean**	86 (22.8%)	56 (22.4%)	142 (22.6%)	(15.7–29.5)
**Type of lactation**	**Maternal**	331 (87.6%)	222 (88.8%)	553 (88.1%)	(85.4–90.8)
	**Artificial**	47 (12.4%)	28 (11.2%)	75 (11.9%)	(4.6–19.2)

## Abbreviations

**VC**	Valencian Community
**ACs**	Autonomous Communities
**TV**	Torrevieja
**EC**	Elx-Crevillent
**AEMPS**	Agencia Española del Medicamento y Productos Sanitarios (Spanish Agency of Medicines and Health Products)
**NVR**	Nominal Vaccination Registry

## References

[1] Kachikis A., Eckert L.O., Englund J. (2018). Who is the Target: Mother or Baby?. Viral Immunol..

[2] World Health Organization Vaccine Position Papers (November 2012). https://www.who.int/wer/2012/wer8747.pdf?ua=1.

[3] Schmid P., Rauber D., Betsch C., Lidolt G., Denker M.L. (2017). Barriers of Influenza Vaccination Intention and Behavior—A Systematic Review of Influenza Vaccine Hesitancy, 2005–2016. PLoS ONE.

[4] Larson H.J., Jarrett C., Eckersberger E., Smith D.M., Paterson P. (2014). Understanding vaccine hesitancy around vaccines and vaccination from a global perspective: A systematic review of published literature, 2007–2012. Vaccine.

[5] Larson H.J., Clarke R.M., Jarrett C., Eckersberger E., Levine Z., Schulz W.S., Paterson P. (2018). Measuring trust in vaccination: A systematic review. Hum. Vaccines Immunother..

[6] Office of Disease Prevention and Health Promotion (2017). Immunization and Infectious Diseases—Healthy People 2020 Topics and Objectives.

[7] Kerr S., Van Bennekom C.M., Mitchell A.A. (2016). Influenza Vaccination Coverage during Pregnancy—Selected Sites, United States, 2005–2006 Through 2013-14 Influenza Vaccine Seasons. MMWR Morb. Mortal. Wkly. Rep..

[8] Groom H.C., Henninger M.L., Smith N., Koppolu P., Cheetham T.C., Glanz J.M., Hambidge S.J., Jackson L.A., Kharbanda E.O., Klein N.P. (2016). Influenza Vaccination During Pregnancy: Influenza Seasons 2002–2012, Vaccine Safety Datalink. Am. J. Prev. Med..

[9] Centers for Disease Control and Prevention (CDC) (2017). Cobertura de Vacunación Contra la Influenza Entre Mujeres Embarazadas, Estados Unidos, Temporada de Influenza 2015–2016. https://espanol.cdc.gov/enes/flu/fluvaxview/pregnant-coverage_1516estimates.htm.

[10] Conselleria de Sanitat (2013). Prevención y Vigilancia de la Gripe en la Comunidad Valenciana Temporada 2012–2013.

[11] Ministerio de Sanidad Servicios Sociales e Igualdad (2019). Coberturas de Vacunación. Datos Estadísticos.

[12] (2014). Guía de Práctica Clínica de Atención en el Embarazo y Puerperio.

[13] Maertens K., De Schutter S., Braeckman T., Baerts L., Van Damme P., De Meester I., Leuridan E. (2014). Breastfeeding after maternal immunisation during pregnancy: Providing immunological protection to the newborn: A review. Vaccine.

[14] Nunes M.C., Madhi S.A. (2018). Prevention of influenza-related illness in young infants by maternal vaccination during pregnancy. F1000Research.

[15] Galvao T.F., Silva M.T., Zimmermann I.R., Lopes L.A.B., Bernardo E.F., Pereira M.G. (2013). Influenza vaccination in pregnant women: A systematic review. ISRN Prev. Med..

[16] Laenen J., Roelants M., Devlieger R., Vandermeulen C. (2015). Influenza and pertussis vaccination coverage in pregnant women. Vaccine.

[17] Odone A., Ferrari A., Spagnoli F., Visciarelli S., Shefer A., Pasquarella C., Signorelli C. (2015). Effectiveness of interventions that apply new media to improve vaccine uptake and vaccine coverage. Hum. Vaccines Immunother..

[18] Barber A., Muscoplat M.H., Fedorowicz A. (2017). Coverage with Tetanus, Diphtheria, and Acellular Pertussis Vaccine and Influenza Vaccine among Pregnant Women—Minnesota, March 2013–December 2014. MMWR Morb. Mortal. Wkly. Rep..

[19] Patten S., Vollman A.R., Manning S.D., Mucenski M., Vidakovich J., Dele Davies H. (2006). Vaccination for Group B Streptococcus during pregnancy: Attitudes and concerns of women and health care providers. Soc. Sci. Med..

[20] Young B., Sadarangani S., Jiang L., Wilder-Smith A., Chen M.I. (2018). Duration of Influenza Vaccine Effectiveness: A Systematic Review, Meta-analysis, and Meta-regression of Test-Negative Design Case-Control Studies. J. Infect. Dis..

[21] Yamyoshi S., Kawaoka Y. (2019). Current and future influenza vaccines. Nat. Med..

[22] Hu Y., Wang Y., Liang H., Chen Y. (2017). Seasonal Influenza Vaccine Acceptance among Pregnant Women in Zhejiang Province, China: Evidence Based on Health Belief Model. Int. J. Environ. Res. Public Health.

[23] McCarthy E.A., Pollock W.E., Tapper L., Sommerville M., McDonald S. (2015). Increasing uptake of influenza vaccine by pregnant women post H1N1 pandemic: A longitudinal study in Melbourne, Australia, 2010 to 2014. BMC Pregnancy Childbirth.

[24] Costantino C., Vitale F. (2016). Influenza vaccination in high-risk groups: A revision of existing guidelines and rationale for an evidence-based preventive strategy. J. Prev. Med. Hyg..

[25] Rodríguez-Blanco N., Tuells J., Vila-Candel R., Nolasco A. (2019). Adherence and Concordance of Influenza and Pertussis Vaccination Coverage in Pregnant Women in Spain. Int. J. Environ. Res. Public Health.

[26] Li T., Lv M., Lei T., Wu J., Pang X., Deng Y., Xie Z. (2016). Who benefits most from influenza vaccination policy: A study among the elderly in Beijing, China?. Int. J. Equity Health.

[27] Conselleria de Sanitat Universal i Salut Pública (2017). Estrategias de Vacunación Frente a la Gripe 2016–2017.

[28] Hofstetter A.M., Durivage N., Vargas C.Y., Camargo S., Vawdrey D.K., Fisher A., Stockwell M.S. (2015). Text message reminders for timely routine MMR vaccination: A randomized controlled trial. Vaccine.

[29] Eiros-Bouza J.M., Perez-Rubio A. (2015). Burden of influenza virus type B and mismatch with the flu vaccine in Spain. Rev. Esp. Quimioter..

[30] Ciancio B.C., Rezza G. (2014). Costs and benefits of influenza vaccination: More evidence, same challenges. BMC Public Health.

[31] Vilca L.M., Verma A., Buckeridge D., Campins M., Yengle L.M.V. (2017). A population-based analysis of predictors of influenza vaccination uptake in pregnant women: The effect of gestational and calendar time. Prev. Med..

[32] Myers K.L. (2016). Predictors of maternal vaccination in the United States: An integrative review of the literature. Vaccine.

[33] Rodríguez-Blanco N., Soriano Monreal C., Vegara López I., Botella Y.M., Barceló D.G., García R.C. (2014). Maternidad y Nuevas Tecnologías: “Paritorios Online”. Tesela.

[34] Baum S., Hitschold T., Becker A., Smola S., Solomayer E., Rody A., Rissland J. (2017). Implementation of the Recommendation to Vaccinate Pregnant Women against Seasonal Influenza-Vaccination Rates and Acceptance. Geburtshilfe Frauenheilkd..

[35] Kahn K.E., Black C.L., Ding H., Williams W.W., Lu P.-J., Fiebelkorn A.P., Havers F., D’Angelo D.V., Ball S., Fink R.V. (2018). Influenza and Tdap Vaccination Coverage Among Pregnant Women—United States, April 2018. MMWR Morb. Mortal. Wkly. Rep..

[36] Ahluwalia I.B., Jamieson D.J., Rasmussen S.A., D’angelo D., Goodman D., Kim H. (2010). Correlates of seasonal influenza vaccine coverage among pregnant women in Georgia and Rhode Island. Obstet. Gynecol..

[37] Donahue J.G., Kieke B.A., King J.P., DeStefano F., Mascola M.A., Irving S.A., Cheetham T.C., Glanz J.M., Jackson L.A., Klein N.P. (2017). Association of spontaneous abortion with receipt of inactivated influenza vaccine containing H1N1pdm09 in 2010–2011 and 2011–2012. Vaccine.

[38] Centers for Disease Control and Prevention (CDC) Pregnant Women and Flu Vaccination, Internet Panel Survey, United States, November 2017. https://www.cdc.gov/flu/fluvaxview/pregnant-women-nov2017.htm.

[39] Napolitano F., Napolitano P., Angelillo I.F. (2017). Seasonal influenza vaccination in pregnant women: Knowledge, attitudes, and behaviors in Italy. BMC Infect. Dis..

[40] Lang A.Y., Boyle J.A., Fitzgerald G.L., Teede H., Mazza D., Moran L.J., Harrison C. (2018). Optimizing preconception health in women of reproductive age. Minerva Ginecol..

[41] Mazagatos C., Delgado-Sanz C., Oliva J., Gherasim A., Larrauri A., Spanish Influenza Surveillance System (2018). Exploring the risk of severe outcomes and the role of seasonal influenza vaccination in pregnant women hospitalized with confirmed influenza, Spain, 2010/11–2015/16. PLoS ONE.

[42] Mertz D., Geraci J., Winkup J., Gessner B.D., Ortiz J.R., Loeb M. (2017). Pregnancy as a risk factor for severe outcomes from influenza virus infection: A systematic review and meta-analysis of observational studies. Vaccine.

[43] Zingg A., Siegrist M. (2012). Measuring people’s knowledge about vaccination: Developing a one-dimensional scale. Vaccine.

[44] Yuen C.Y., Tarrant M. (2014). Determinants of uptake of influenza vaccination among pregnant women—A systematic review. Vaccine.

[45] Ishola D.A., Permalloo N., Cordery R.J., Anderson S.R. (2013). Midwives’ influenza vaccine uptake and their views on vaccination of pregnant women. J. Public Health.

[46] Morgan J.L., Baggari S.R., Chung W., Ritch J., McIntire D.D., Sheffield J.S. (2015). Association of a Best-Practice Alert and Prenatal Administration with Tetanus Toxoid, Reduced Diphtheria Toxoid, and Acellular Pertussis Vaccination Rates. Obstet. Gynecol..

[47] Wong V.W., Lok K.Y., Tarrant M. (2016). Interventions to increase the uptake of seasonal influenza vaccination among pregnant women: A systematic review. Vaccine.

[48] Kharbanda E.O., Vargas C.Y., Castaño P.M., Lara M., Andres R., Stockwell M.S. (2011). Exploring pregnant women’s views on influenza vaccination and educational text messages. Prev. Med..

[49] Bödeker B., Betsch C., Wichmann O. (2015). Skewed risk perceptions in pregnant women: The case of influenza vaccination. BMC Public Health.

[50] Getahun D., Fassett M.J., Peltier M.R., Takhar H.S., Shaw S.F., Im T.M., Chiu V.Y., Jacobsen S.J. (2019). Association between seasonal influenza vaccination with pre- and postnatal outcomes. Vaccine.

[51] Nunes M.C., Madhi S.A. (2017). Influenza vaccination during pregnancy for prevention of influenza confirmed illness in the infants: A systematic review and meta-analysis. Hum. Vaccines Immunother..

[52] Wiley K.E., Cooper S.C., Wood N., Leask J. (2015). Understanding pregnant women’s attitudes and behavior toward influenza and pertussis vaccination. Qual. Health Res..

[53] Meharry P.M., Colson E.R., Grizas A.P., Stiller R., Vázquez M. (2013). Reasons why women accept or reject the trivalent inactivated influenza vaccine (TIV) during pregnancy. Matern. Child Health J..

[54] Jung M. (2018). The effect of maternal decisional authority on children’s vaccination in East Asia. PLoS ONE.

[55] Simaku A., Preza E., Nelaj J., Sulo J., Bino S. (2019). Knowledge, attitudes, and practice regarding influenza vaccination in pregnant women in Albania. Int. J. Infect. Dis..

[56] World Health Organization Ten Threats to Global Health in 2019. https://www.who.int/emergencies/ten-threats-to-global-health-in-2019.

